# Chronic Electroconvulsive Shock Disrupts Hippocampal Physiology and Impairs Associative Memory in Mice

**DOI:** 10.1002/hipo.70142

**Published:** 2026-10-14

**Authors:** T. R. Zhang, N. P. Vyleta, I. Schwein, B. Askari, M. B. Cooke, F. Vila‐Rodriguez, J. S. Snyder

**Affiliations:** ^1^ Department of Psychology University of British Columbia Vancouver Canada; ^2^ Djavad Mowafaghian Centre for Brain Health University of British Columbia Vancouver Canada; ^3^ Non‐Invasive Neurostimulation Therapies Laboratory, Department of Psychiatry, Faculty of Medicine University of British Columbia Vancouver Canada; ^4^ School of Biomedical Engineering University of British Columbia Vancouver Canada

## Abstract

Electroconvulsive therapy (ECT) is a highly effective treatment for depression but it has undesirable side effects on various aspects of cognition, including memory. Patients may display retrograde amnesia for autobiographical events and anterograde amnesia in tests of recall and recognition memory. These amnestic effects are likely to depend, at least in part, on changes in the hippocampus. ECT‐induced volume increases in the hippocampus and dentate gyrus subregion in particular correlate with the cognitive deficits. However, the precise cellular and behavioral effects of ECT remain poorly understood. Here, we therefore subjected male and female mice to chronic electroconvulsive shock (ECS) and examined effects on hippocampal physiology and memory. Perforant path inputs onto dentate gyrus neurons were examined using whole cell patch clamp recordings in hippocampal slices. In the intact slice, perforant path stimulation did not result in any synaptic or spiking differences in mice given sham versus ECS treatments. However, GABA_A_ blockade increased EPSPs and spiking in sham, but not ECS‐treated, mice. Voltage clamp recordings further revealed weaker perforant path excitation of dentate gyrus neurons in ECS‐treated mice. Extrapolating these findings to the intact animal, we then used Fos expression as a proxy for neural activity and found that ECS blunted exploration‐induced hippocampal activation, which was most pronounced in the dentate gyrus. Finally, recognition memory tasks revealed that ECS impaired memory for object‐context associations but not memory for objects or locations. Collectively, these experiments reveal that a clinically relevant schedule of ECS disrupts synaptic and circuit functions of the hippocampus and causes anterograde amnesia in a test of episodic‐like associative memory.

## 1 Introduction

Electroconvulsive therapy (ECT) is the most effective treatment for severe depressive disorder (Group TUER 2003) but cognitive side effects may deter patients from pursuing it as a treatment option (Koopowitz et al. 2003; Kirov et al. 2016). A major goal is therefore to clarify the neurobiological mechanisms so that procedural refinements can maintain the therapeutic efficacy while minimizing unwanted side effects (Deng et al. 2024).

ECT's broad effects on cognition are apparent anywhere from hours to months after treatment depending on the task and behavior being tested. Meta analyses indicate cognitive deficits are primarily observed in the days following treatment and that, weeks later, there is recovery and/or improvement (Semkovska and McLoughlin 2010; Landry et al. 2020). One pattern of initial deficits suggests disruption to the hippocampus and medial temporal lobe systems involved in episodic learning and memory. For example, patients might display retrograde amnesia for autobiographical or public events that occurred prior to treatment (Squire et al. 1975; Lisanby et al. 2000). They also display transient impairments in anterograde recognition memory for discrete items (Squire and Miller 1974; Steif et al. 1986; Sackeim et al. 2000; Falconer et al. 2010; Fernie et al. 2014). However, recognition memory performance can be ascribed to hippocampal‐dependent recollection or neocortex‐dependent familiarity judgments (Eichenbaum et al. 2007) though, to date, ECT studies have not differentiated between these 2 processes. Since ECT increases in hippocampal volume are prominent (van Oostrom et al. 2018; Gbyl et al. 2020; Argyelan et al. 2021) and associated with cognition and memory impairments (van Oostrom et al. 2018; Argyelan et al. 2021), one might expect preferential disruption to hippocampal‐dependent associative recognition memory over recognition memory for discrete items.

In human studies it is difficult to probe the neurobiological consequences of ECT beyond the level of volumetric changes. Animal models of electroconvulsive shock (ECS) are therefore helpful for identifying how clinically‐relevant schedules of ECS impact cellular and circuit functions. Subregional analyses indicate that DG volume increases are particularly robust and are most closely linked to the cognitive deficits (Takamiya et al. 2019; Nuninga et al. 2020; Gbyl et al. 2021). Consistent with volumetric increases in the DG after ECT, ECS in rodents potently upregulates the production of newborn neurons in the adult DG (Madsen et al. 2000; Malberg et al. 2000; Perera et al. 2007; Ueno et al. 2019; Zhang, Guilherme, et al. 2021) and promotes their morphological growth (Zhang et al. 2026). ECS can reverse long‐term potentiation, suggesting it may be capable of disrupting previously‐encoded memories (Hesse and Teyler 1976). Furthermore, it has been found to increase synaptic strength in the hippocampus and reduce the capacity for subsequent long‐term potentiation, aligning with deficits in anterograde learning and memory processes (Stewart et al. 1994; Stewart and Reid 2000; Chen et al. 2018). Nonetheless, many details remain unknown about how ECS impacts excitatory and inhibitory features of hippocampal circuitry.

To address these gaps, here we subjected mice to chronic ECS and examined DG synaptic transmission and spiking in hippocampal slices, in vivo activation (Fos expression) of the hippocampus during learning, and learning and memory in associative and non‐associative recognition memory paradigms. These experiments revealed reduced strength of cortical input synapses, blunted expression of the immediate‐early gene Fos, and impairments in associative recognition memory. Collectively, these data identify novel, behaviorally‐relevant neurobiological consequences of ECS that may be useful for understanding the off‐target effects of ECT in humans.

## 2 Methods

### 2.1 Animals and General Experimental Design

All procedures were approved by the Animal Care Committee at the University of British Columbia and conducted in accordance with the Canadian Council on Animal Care guidelines. The mice used in this study were Cre‐negative animals resulting from a cross between Ascl1^CreERT2^ mice (Kim et al. 2011) and Ai14 mice (Madisen et al. 2010), which were being used for a separate study. We opted to use the Cre‐negative littermates for this study to reduce the number of animals used and because both parent lines are on a C57Bl/6J background, which is commonly used in studies of behavior and hippocampal function. Mice were weaned at 21 days of age into cages of 2–4 mice where they had *ad libitum* access to food and water and were housed on a 12 h light/dark cycle with lights on at 7 am. At 2–3 months old, mice were given ECS or sham stimulation. Animals received sham stimulation or ECS every 2 days for a total of 10 sessions over 20 days. Behavior testing began 2 days after the last stimulation session.

### 2.2 Electroconvulsive Shock

Animals were handled daily for 1 week before ECS to minimize stress associated with manipulation. On each stimulation day, a single animal was removed from its home cage and transferred to an induction chamber containing 5% isoflurane delivered in 20% oxygen. When the animal no longer showed the ability to right itself, anesthesia was reduced and maintained at 2%. The animal was then positioned on a wire platform that supported the body while allowing unrestricted limb movement. Isoflurane (2%) was continuously administered via a nose cone, and a toe‐pinch reflex test was performed to confirm adequate anesthesia. After verifying loss of reflexes, both ears were wiped with 70% isopropyl alcohol followed by saline to improve electrical conductivity. Ear clip electrodes were attached, and a conductance test was run to ensure proper contact. ECS was delivered at 30 mA, 100 Hz, with a 0.5‐ms square pulse width for a total train duration of 1 s to elicit a tonic–clonic seizure. If an animal showed habituation to stimulation in later sessions, the train duration was increased to 1.2 s and then to 1.5 s as needed to induce a seizure. This within‐session titration of the stimulus strength mimics dosing protocols used in the clinic, which ensures that a therapeutic seizure is elicited (Davies and Wilson 2001). In total, for the behavior Experiment 1 mouse received up to 1.5 s and for the Fos Experiment 2 mice received 1.2 s and 1 mouse received 1.5 s. Records for the electrophysiological experiment were unavailable but these mice were treated similarly. No animal required more than 1.5 s to induce a seizure. After seizure activity had ceased for at least 1 min, the animal was transferred to a warmed recovery cage and monitored until normal behavior returned, after which it was placed back in its home cage. While repeated isoflurane exposure may alter hippocampal plasticity and behavior, these adverse effects are typically observed following longer exposures during juvenility and not adulthood (Zhu et al. 2009; Stratmann et al. 2010). Importantly, patients are also administered ECT while under anesthesia, and sham mice underwent identical procedures except that, following electrode placement, they remained under isoflurane for 2 min without receiving any electrical stimulation. Thus, group differences reflect those caused by ECS‐induced seizures and not anesthesia.

### 2.3 Behavioral Testing

Mice were habituated to an open field box (40 × 40 × 40 cm opaque box) on day 2 after the last session of ECS. Habituation consisted of animals being put in the open field arena without any objects for 10 min.

On day 3, the animals were trained and tested on a novel object recognition test. In the training phase, animals were put into the familiar open field box with 2 identical objects for 10 min. An hour after training, the animals were put back into the box, now with a familiar object and a novel object. The testing phase lasted 5 min. Objects were counter‐balanced for familiarity and novelty across animals.

On day 4, mice were tested for novel place recognition in the same arena as used to test object recognition. The procedure was the same as novel object recognition except the location, and not identity, of one of the objects changed in the testing phase.

On day 5, mice were tested on novel object‐context recognition. Here, mice were placed into two consecutive novel contexts that differed from the one used for object and place recognition in that the walls and floor of the open field box were replaced with materials of different texture and color. New scents were added to the boxes by taping a paper towel with a diluted scent onto the outside walls of the boxes (coffee and vanilla, diluted 1:50 with tap water). In the first context, mice explored a pair of objects (AA) for 10 min. After a 3‐min interval in their home cage, mice explored another pair of objects (BB) in the second context. An hour after the second training phase, the animals were put back in the first context and explored 2 objects, one of which was previously seen in that context (A, familiar), and another that had been previously seen but not in that context (B, novel) for 5 min.

For behavioral induction of Fos expression, behaviorally naïve mice were allowed to explore the open field box for 10 min, 5 days after chronic ECS treatment. They were then perfused 60 min later and brains were collected for histological processing.

### 2.4 Tissue Preparation and Immunohistochemistry

Mice were transcardially perfused with 4% paraformaldehyde in phosphate buffered saline and brains were post‐fixed for an additional 24 h. Brains were then transferred to a glycerol‐based cryoprotectant solution, and a 1‐in‐6 series of 40 μm coronal sections spanning the hippocampus were cut on an AO860 freezing microtome. For free floating fluorescent immunostaining of c‐fos, sections were incubated with 1% H_2_O_2_ for 30 min, rabbit anti‐Fos antibody (1:1000; Synaptic Systems) for 3 days at 4°C, biotinylated goat anti‐rabbit secondary antibody (Jackson Immunoresearch; 1:250) for 1 h at room temperature, streptavidin‐HRP for 1 h and finally streptavidin‐conjugated Alexa 647 (Thermofisher). Slices were then stained with DAPI, mounted onto slides and cover‐slipped with PVA‐DABCO.

### 2.5 Imaging and Analyses

All images were acquired with a Leica SP8 confocal microscope. Both dorsal and ventral hippocampal slices were analyzed for all measures. Images of Fos expression were acquired with a 40X oil immersion lens at 1.0 zoom. Images of 1024 × 1024 pixels (400 Hz scan speed) in size at a *z*‐resolution of 1.5 μm were merged to encompass the entire hippocampus. Four sections from each animal were chosen (2 dorsal and 2 ventral sections, spaced ≥ 240 μm apart; dorsal Bregma −1.8 to −2.2 mm, ventral −3.0 to −3.8 mm) and all Fos^+^ cells were counted on ImageJ using the Cell Counter plugin. Cell densities (in mm^2^) were then obtained by dividing cell counts by the analyzed area of the granule cell and pyramidal cell layers.

### 2.6 Electrophysiology

Within 1 week after the end of a session of chronic ECS, mice were anesthetized with sodium pentobarbital (50 mg/kg, I.P.) and perfused with ice‐cold cutting solution containing (in mM): 93 NMDG, 2.5 KCl, 1.2 NaH_2_PO_4_, 30 NaHCO_3_, 20 HEPES, 25 glucose, 5 sodium ascorbate, 3 sodium pyruvate, 10 n‐acetyl cysteine, 0.5 CaCl_2_, 10 MgCl_2_ (pH‐adjusted to 7.4 with HCl and equilibrated with 95% O_2_ and 5% CO_2_, ~310 mOsm). Hippocampal slices were cut on a vibratome and transferred to the cutting solution at 35°C for 20 min, and then transferred to a storage solution containing (in mM): 87 NaCl, 25 NaHCO_3_, 2.5 KCl, 1.25 NaH_2_PO_4_, 10 glucose, 75 sucrose, 0.5 CaCl_2_, 7 MgCl_2_ (equilibrated with 95% O_2_ and 5% CO_2_, ~325 mOsm) at 35°C prior to starting experiments.

At ~32°C, whole‐cell patch‐clamp recordings were made from unlabelled granule cells that were located in the middle of the granule cell layer in the suprapyramidal blade of the dentate gyrus. Slices were superfused with an artificial cerebrospinal fluid (ACSF) containing (in mM): 125 NaCl, 25 NaHCO_3_, 2.5 KCl, 1.25 NaH_2_PO_4_, 25 glucose, 1.2 CaCl_2_, 1 MgCl_2_ (equilibrated with 95% O_2_ and 5% CO_2_, ~320 mOsm). Recording pipettes were made from 2.0 mm/1.16 mM (OD/ID) borosilicate glass capillaries and had resistance ~5 MΩ with an internal solution containing (in mM): 120 K‐gluconate, 15 KCl, 2 MgATP, 10 HEPES, 10 EGTA, 0.3 Na_2_GTP, 7 Na_2_‐phosphocreatine (pH 7.28 with KOH, ~300 mOsm). Current‐clamp and voltage‐clamp recordings were performed at −80 mV. Only recordings with high seal resistance (several giga‐ohms) and low holding current (less than 50 pA) were included in analyses. For current‐clamp recordings, series resistance and pipette capacitance were compensated with the bridge balance and capacitance neutralization circuits of the amplifier.

We first conducted recordings in current‐clamp to determine intrinsic properties of granule cells (resting membrane potential, input resistance, and rheobase, that is, minimum current injection needed to elicit spiking). Synaptic and spiking properties were then evaluated in response to medial perforant path stimulation, using trains of stimuli (10 Hz, 1 s). Stimuli (0.1 ms) were delivered through a stimulus isolator (A‐M Systems analog stimulus isolator model 2200). In ACSF, 10 trains were delivered using stimulus intensities from 100 to 1000 μA. Synaptic input–output curves were generated by measuring excitatory post‐synaptic potential (EPSP) size in response to the first pulse of each train. Spiking was measured over all 10 pulses of each train. The same slices were then superfused with the GABA_A_ blocker, bicuculine methiodide (10 μM), and the same stimulus trains were again delivered to determine the extent to which input–output curves and spiking were constrained by GABAergic inhibition. We then switched to a voltage clamp configuration and measured synaptic currents elicited by medial perforant path stimulation at 100–1000 μA.

### 2.7 Behavioral Analysis

For the habituation session, Deeplabcut (Mathis et al. 2018) was used to quantify distance traveled and time spent in the center 10 × 10 cm zone, as measures of activity and anxiety‐related behaviors, respectively. Distance traveled was similarly measured for the sample and test phases of the object recognition tests.

Object exploration behaviors were manually scored by the experimenter. The frequency and duration of object exploration events were quantified, where an exploration event was defined as direct sniffing or interacting with an object, not including time spent on top of the object looking around the arena.

Recognition memory was assessed in 2 ways. First, we directly compared the absolute amount of exploration of novel versus familiar stimuli. Second, we calculated novelty‐based exploration biases as discrimination indices, defined as (Exploration_NOVEL_—Exploration_FAMILIAR_)/Exploration_NOVEL+FAMILIAR_. A positive discrimination index indicates that the animal explored the novel object (or object configuration) more often/for a longer period than the familiar object and is interpreted as object memory or preference. A discrimination index of 0 results from equivalent investigation of the 2 objects, indicating a lack of memory or object preference.

### 2.8 Statistical Analyses

All underlying data are provided as Supporting Information (“hipo70142‐sup‐0001‐DataS1.xlsx”). All data collection was performed blind to the treatment collection of the animal. Sample sizes for each experiment are specified in the results section. While we did not perform formal power calculations, sample sizes were chosen based on our prior work that has detected moderate group effects in DG physiology (Vyleta and Snyder 2021; Ash et al. 2025), DG Fos^+^ cell counts (Snyder et al. 2009), and object recognition behavior (Seib et al. 2018). Statistical analyses were performed using R and Prism 10 (Graphpad) and graphs were prepared with Prism 10. Sample sizes for each experiment are indicated in the results section. Electrophysiological membrane property data were pooled across sexes since sample sizes were too small to include sex as a factor, and analyzed by *t*‐test. Synaptic transmission and spiking data were fit with linear mixed‐effects models with treatment (sham and ECS), sex (male and female), stimulus intensity (100–1000 μA) and drug (ACSF and bicuculline, except the excitatory post‐synaptic current (EPSC) experiment, which was conducted entirely in bicuculline) as fixed effects and cell (as appropriate), nested within mouse, as a random effect. The data were then analyzed by ANOVA. Three sham cells and 1 ECS cell were excluded from the EPSP analyses because they spiked in the presence of bicuculline, thereby obscuring the synaptic response. For recognition memory tasks, repeated measures ANOVA was used to analyze effects of ECS on exploration of novel versus familiar stimuli. Discrimination indices were also analyzed by ANOVA (treatment × sex). Two sham‐treated mice were excluded from the object recognition analyses because they failed to explore the objects during the tests. Locomotor behavior was analyzed by *t*‐test (habituation) and mixed‐effects models (object recognition) and sexes were pooled because there were insufficient numbers of both sexes (in part because some videos were not suitable for automated tracking with Deeplabcut). In all analyses, significant interactions were followed up with post hoc Holm‐Sidak tests. For all analyses, significance was defined as α < 0.05.

## 3 Results

### 3.1 ECS Does Not Alter Intrinsic Membrane Properties of DG Granule Cells

To test whether ECS caused physiological changes in DG granule cells we performed whole cell patch clamp recordings from neurons located in the middle of the granule cell layer. Intrinsic membrane properties were measured from cells from sham and ECS‐treated mice and the sexes were pooled due to limited sample sizes (female sham: 5 cells from 2 mice; male sham: 7 cells from 3 mice; female ECS: 11 cells from 4 mice, male ECS: 11 cells from 4 mice). For all measures, there was no effect of ECS on the membrane properties of DG neurons (Figure 1a–c): ECS‐treated mice had normal resting membrane potential (*T*_32_ = 0.3, *p* = 0.8), input resistance (*T*_32_ = 0.7, *p* = 0.7) and the minimum current needed to elicit an action potential was not different between sham‐ and ECS‐treated mice (*T*_32_ = 1.1, *p* = 0.3).

**FIGURE 1:**
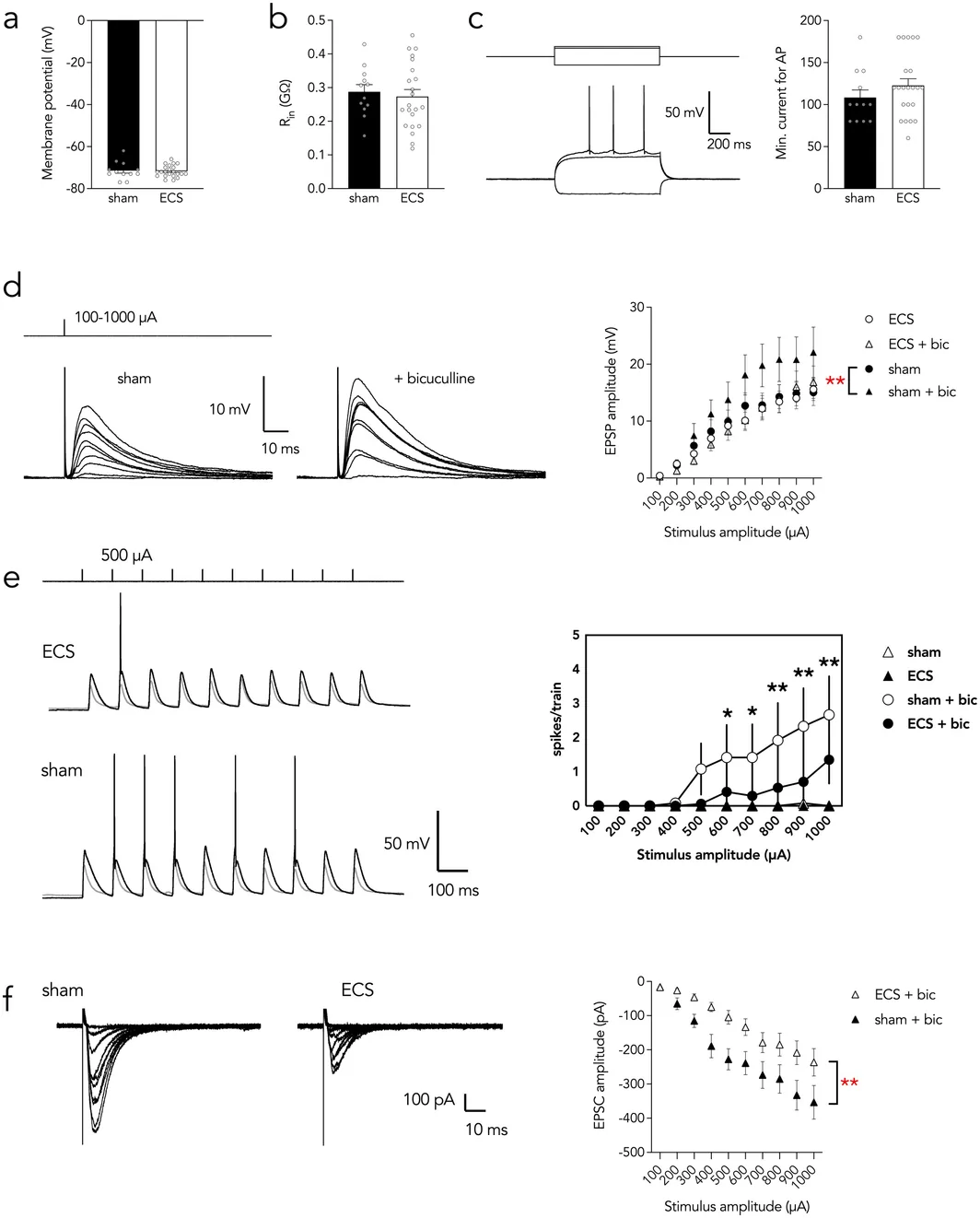
Weaker excitatory synapses and less GABAergic modulation in mice treated with ECS. (a) ECS did not alter the resting membrane potential of granule cells. (b) ECS did not alter input resistance. (c) ECS did not alter the minimum current needed to elicit an action potential. (d) GABA_A_ blockade, with bicuculline, increased EPSP magnitude in sham‐treated mice but not ECS‐treated mice. (e) GABA_A_ blockade increased spiking in sham‐treated, but not ECS‐treated, mice at higher stimulus intensities. Gray traces are voltage recordings (primarily EPSPs) in ACSF and black traces are recordings in bicuculline, which display higher levels of spiking. (f) Under GABA_A_ blockade, sham‐treated mice had larger EPSCs than ECS‐treated mice. Bars and symbols indicate mean ± standard error. *p < 0.05, **p < 0.01.

### 3.2 ECS Reduces Synaptic Activation of DG Granule Neurons

We next examined stimulus–response characteristics at medial perforant path inputs in both ACSF and in the presence of bicuculline, a GABA_A_ blocker (cells from sham mice: 5 females, 4 males; ECS mice: 9 females, 7 males). Afferent trains of stimuli (10 pulses at 10 Hz, 100–1000 μA) were delivered and EPSPs and postsynaptic spiking were recorded. The magnitude of the first EPSP of the train was used to assess baseline synaptic strength. As expected, synaptic responses increased with greater stimulus amplitude (*F*_9,29_ = 4, *p* = 0.001) and in the presence of bicuculline (*F*_1,7_ = 10, *p* = 0.02). There was no main effect of ECS treatment (*F*_1,8_ = 0.9, *p* = 0.4) or sex (*F*_1,8_ = 0.7, *p* = 0.4) but there was an ECS × bicuculline interaction (*F*_1,7_ = 11, *p* = 0.01). Post hoc comparisons revealed that synaptic potentials did not differ between sham‐ and ECS‐treated mice in ACSF. In contrast, in the presence of the GABA_A_ blocker bicuculline, EPSPs were larger in sham mice than in ECS mice (Figure 1d; sham mice: ACSF vs. bicuculline: *p* = 0.008; ECS mice: ACSF vs. bicuculline: *p* = 0.9). All other interactions were not significant (all *Ps* > 0.06).

Since inhibition profoundly shapes perforant path‐DG coupling (Ewell and Jones 2010; Marín‐Burgin et al. 2012), we also measured spiking during the trains of afferent stimulation. As described above, stimulation was performed in ACSF, with GABA intact, and then repeated in the presence of the GABA_A_ blocker, bicuculline (Figure 1e; cells from sham mice: 5 females, 7 males; ECS mice: 10 females, 7 males). There were no main effects of stimulus intensity (*F*_9,26_ = 0.9, *p* = 0.5), bicuculline (*F*_1,7_ = 4, *p* = 0.09), ECS treatment (ECS: *F*_1,13_ = 4, *p* = 0.08) or sex (*F*_1,13_ = 0, *p* = 0.9). Consistent with prior work, mature DG neurons rarely spiked in ACSF but increased spiking during GABA_A_ blockade (Marín‐Burgin et al. 2012) (averaging ~1–3 spikes/train; ECS × bicuculline interaction: *F*_9,425_ = 10, *p* < 0.0001). Furthermore, a 3‐way interaction between stimulus intensity, bicuculline and ECS suggested that inhibition differentially modulated spiking in sham versus ECS mice (*F*_9,425_ = 3, *p* = 0.007; all other interactions *p* > 0.2). We therefore performed pairwise comparisons of spiking in ACSF versus bicuculline, at each of the stimulation intensities, within the sham‐ and ECS‐treated groups. This revealed that GABA_A_ blockade increased spiking in sham, but not ECS, mice at higher stimulus intensities (600–1000 μA; Figure 1e). Collectively, these results indicate that ECS alters excitation‐inhibition balance in the DG and reduces synaptic excitation of DG granule neurons under GABA_A_ blockade.

To further probe ECS effects on excitatory synaptic transmission, we recorded EPSCs in response to medial perforant path stimulation in the presence of bicuculline (Figure 1f; cells from sham mice: 5 females, 7 males; ECS mice: 10 females, 6 males). As expected, there was a significant effect of stimulation intensity, reflecting the larger EPSCs at higher levels of stimulation (*F*_9,231_ = 22, *p* < 0.0001). There were no differences between males and females (*F*_1,8_ = 0.3, *p* = 0.6) and no significant interactions involving sex (all *Ps* > 0.1). However, ECS‐treated mice had substantially smaller EPSCs, indicating that ECS directly reduces excitatory synaptic strength in the DG (effect of ECS: *F*_1,8_ = 13, *p* = 0.006).

### 3.3 Chronic ECS Reduces Exploration‐Induced Fos Expression

We next tested whether chronic ECS has any effects on neuronal recruitment in vivo, as measured by expression of the experience‐dependent plasticity‐related protein Fos (sham mice: 4 males and 4 females; ECS mice: 4 males and 4 females). Following free exploration in an open field, ECS‐treated mice had blunted Fos expression in both the dorsal and ventral hippocampus (Figure 2). While there was no significant interaction between treatment and DG/CA3/CA1 subregions, Fos reduction was most dramatic in the DG (pooled across dorsal and ventral hippocampus, the overall reduction was 59% in DG, 28% in CA3, 6% in CA1). In the dorsal hippocampus, the DG also had the lowest levels of Fos expression (treatment effect: *F*_1,12_ = 5.2, *p* = 0.04, subregion effect: *F*_1.3,15.9_ = 14, *p* = 0.0009, sex effect: *F*_1,12_ = 0, *p* = 0.8; treatment × subregion interaction: *F*_1.3,15.9_ = 3, *p* = 0.1; treatment × sex interaction: *F*_1,12_ = 0, *p* = 1; treatment × subregion × sex interaction: *F*_1.3,15.9_ = 0.4, *p* = 0.6; DG vs. CA3, *p* = 0.005; DG vs. CA1, *p* = 0.002; CA3 vs. CA1, *p* = 0.05). In the ventral hippocampus, in addition to reduced Fos expression in ECS‐treated mice, there was also greater Fos in females than in males, and there were differences between all hippocampal subregions (DG < CA3 < CA1; treatment effect: *F*_1,12_ = 4.9, *p* = 0.048, subregion effect: *F*_1.6,18.8_ = 28, *p* < 0.0001, sex effect: *F*_1,12_ = 5.9, *p* = 0.03; treatment × subregion interaction: *F*_1.6,18.8_ = 0.1, *p* = 0.9; treatment × sex interaction: *F*_1,12_ = 1.6, *p* = 0.2; treatment × subregion × sex interaction: *F*_1.6,18.8_ = 0.4, *p* = 0.6; DG vs. CA3, *p* = 0.03; DG vs. CA1, *p* < 0.0001; CA3 vs. CA1, *p* = 0.0004).

**FIGURE 2:**
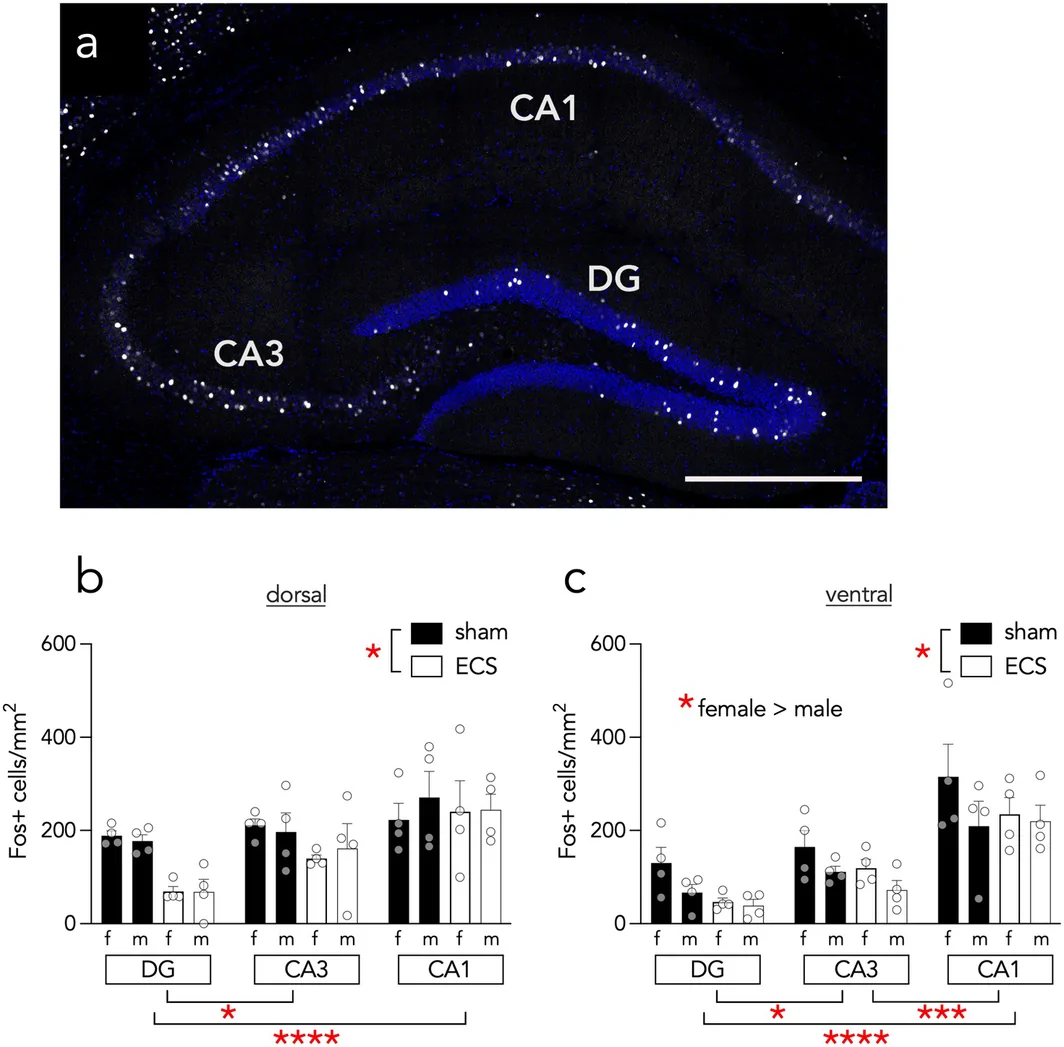
Blunted exploration‐induced Fos expression following chronic ECS. (a) Representative Fos immunostaining across hippocampal subregions (Fos = white, DAPI = blue, scale bar = 500 μm). (b) Five days following chronic ECS, mice showed reduced exploration‐induced Fos expression in the dorsal hippocampus compared to sham‐treated mice. Fos expression was also lower in the DG. (c) ECS‐treated mice also showed reduced exploration‐induced Fos expression in the ventral hippocampus compared to sham‐treated mice. Females has more Fos^+^ cells and all hippocampal subregions differed (DG < CA3 < CA1). Bars indicate mean ± standard error and symbols represent individual mice. *p < 0.05, ***p < 0.001, ****p < 0.0001.

### 3.4 ECS Impairs Associative Recognition Memory

We finally tested whether ECS‐treated mice are impaired on recognition memory tasks that parallel those used to identify anterograde memory deficits in ECT‐treated patients (Squire and Miller 1974; Steif et al. 1986; Falconer et al. 2010; Fernie et al. 2014). During habituation to the open field, prior to memory testing, sham and ECS mice traversed similar distances and spent similar amounts of time in the center of the box indicating comparable levels of activity and innate fear of open spaces, respectively (Figure 3a–c; distance: *T*_26_ = 1.5, *p* = 0.2 L center‐time: *T*_26_ = 0.5, *p* = 0.6). For memory testing, mice were subjected to tests of novel object, novel place and novel object‐context recognition (sham mice: 10 males, 7 females; ECS mice: 10 males, 5 females. For object recognition 1 sham male was excluded due to a lack of object exploration). Within‐subjects analyses of the duration and frequency of exploration events was initially performed by 3‐way repeated measures ANOVA (treatment × sex × object novelty). However, since there were no significant main effects of sex or interactions involving sex (all *p* > 0.05), and to improve the clarity of the figures, we pooled the sexes and performed 2‐way (treatment × object novelty) ANOVAs. We have included sex in the discrimination index graphs, however, to show that similar patterns were generally present in males and females. Additionally, the full dataset (i.e., including breakdown by sex) is available as Supporting Information (“hipo70142‐sup‐0001‐DataS1.xlsx”).

**FIGURE 3:**
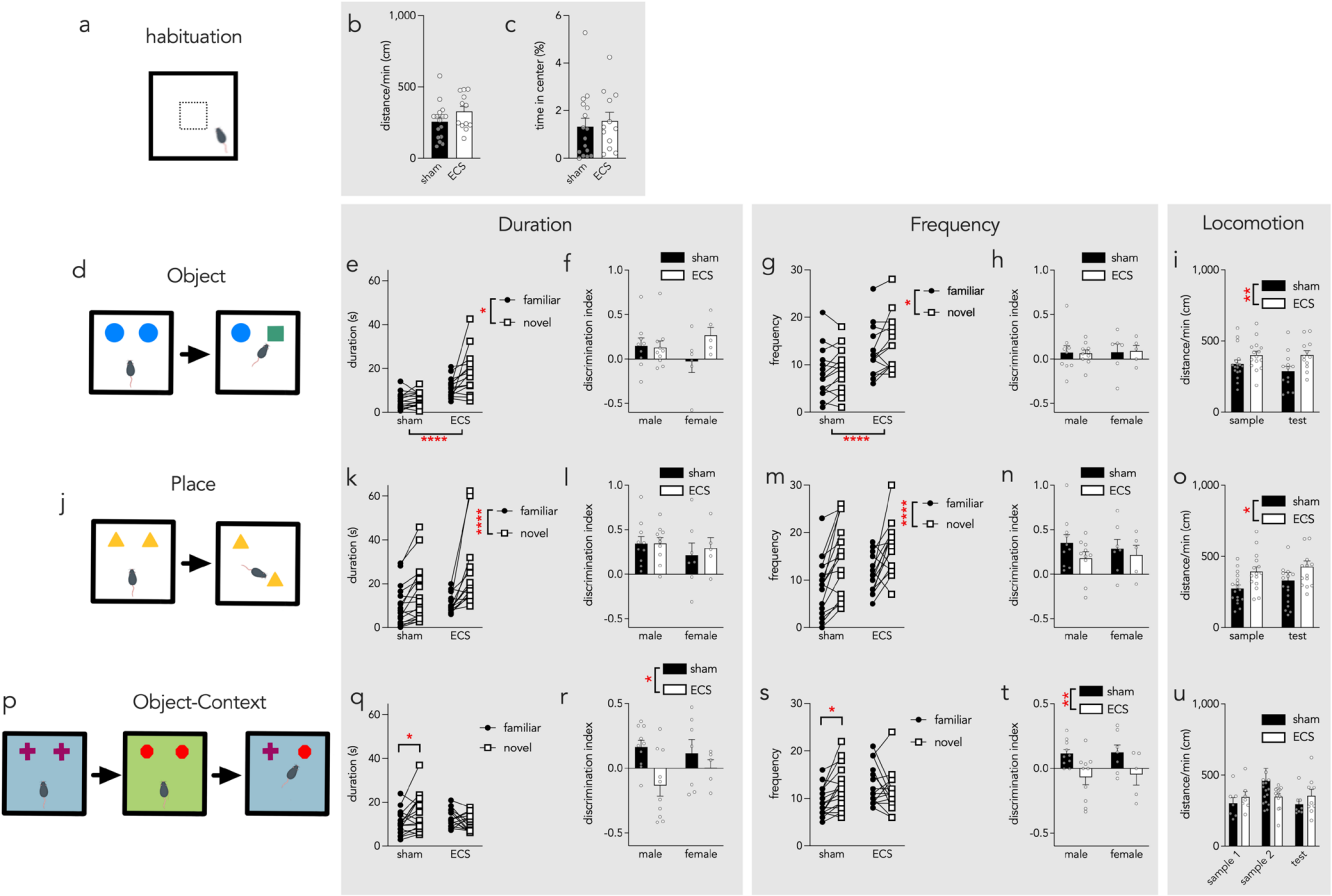
Associative recognition memory is disrupted following ECS. (a–c) Open field habituation. (a) Schematic showing the open field and the 10 × 10 cm center zone. (b) ECS did not alter the distance traveled in the habituation session. (c) ECS did not alter the amount of time spent in the center of the open field during the habituation session. (d–i) Object recognition memory. (d) Object recognition task schematic. Preferential exploration of a novel object is interpreted as memory. (e) At test, ECS‐treated mice spent more time exploring objects and, overall, mice spend more time exploring the novel object. (f) Duration discrimination indices did not differ between groups. (g) Overall, more exploration events were directed towards the novel object and ECS‐treated mice displayed more exploration events. (h) Sham and ECS‐treated mice did not have different discrimination indices. (i) ECS‐treated mice traveled a greated distance than sham‐treated mice. (j–o) Place recognition task. Preferential exploration of the novel location is interpreted as place memory. (k) Both groups spent more time exploring the novel object location. (l) Both groups had comparable, above chance, duration‐based discrimination indices. (m) Both groups had more exploration events directed towards the novel location. (n) Exploration frequency discrimination indices were equivalent between sexes and treatment groups. (o) ECS‐treated mice displayed more locomotion. (p–u) Object‐context recognition task. Preferential exploration of the novel object‐context configuration is interpreted as memory. (q) Sham‐treated, but not ECS‐treated, mice spent more time exploring the novel object‐context configuration. (r) The sham group's duration discrimination index was greater than ECS‐treated mice. (s) Sham‐treated mice directed more exploration events towards the novel object‐context configuration. (t) The sham group's frequency discrimination index was greater than that of the ECS‐treated mice. (u) ECS did not alter locomotor behavior. Bars indicate mean ± standard error. Symbols indicate individual mice. *p < 0.05, **p < 0.01, ****p < 0.0001.

Overall, mice displayed weak, though statistically significant, object recognition memory (Figure 3d–h). Mice spent more time exploring the novel object and directed more exploration events towards the novel object (effect of object on duration: *F*_1,28_ = 5, *p* = 0.03, effect of object on exploration frequency: *F*_1,28_ = 7, *p* = 0.01). ECS‐treated mice also displayed generally greater exploration of the objects regardless of whether they were novel or familiar (effect of ECS on duration: *F*_1,28_ = 31, *p* < 0.0001, effect of ECS on frequency: *F*_1,28_ = 7, *p* = 0.02). However, the treatment × object interactions were not significant, suggesting that ECS does not alter object recognition memory (duration: *F*_1,28_ = 3, *p* = 0.09; frequency: *F*_1,28_ = 0.7, *p* = 0.4). Analyses of the discrimination indices also revealed that neither ECS nor sex altered object recognition memory (*duration*: effect of sex: *F*_1,26_ = 0, *p* = 0.9; effect of ECS: *F*_1,26_ = 2, *p* = 0.2; interaction: *F*_1,26_ = 3, *p* = 0.1; *frequency*: effect of sex: *F*_1,26_ = 0, *p* = 0.8; effect of ECS: *F*_1,26_ = 0, *p* = 1; interaction: *F*_1,26_ = 0, *p* = 0.9). Consistent with the greater levels of overall object exploration, ECS‐treated mice traversed greater distances in the sample and test phases of the novel object recognition task (Figure 3i; effect of testing phase: *F*_1,23_ = 0.7, *p* = 0.4; effect of ECS: *F*_1,30_ = 8, *p* = 0.008; interaction: *F*_1,23_ = 0.8, *p* = 0.8).

In the test of place recognition, sham‐ and ECS‐treated mice showed strong and comparable memory in terms of duration and frequency of exploration events (Figure 3j–n). Furthermore, sham‐ and ECS‐treated mice spent a comparable time, overall, exploring the objects (*duration*: effect of object: *F*_1,30_ = 24, *p* < 0.0001, effect of ECS: *F*_1,30_ = 2, *p* = 0.2, object × ECS interaction: *F*_1,30_ = 4, *p* = 0.05; *frequency*: effect of object: *F*_1,30_ = 31, *p* < 0.0001, effect of ECS: *F*_1,30_ = 1, *p* = 0.3, object × ECS interaction: *F*_1,30_ = 0, *p* = 0.8). Discrimination indices revealed a similar pattern, where both groups displayed strong memory and did not differ from one another (*duration*: effect of sex: *F*_1,28_ = 0.9, *p* = 0.4; effect of ECS: *F*_1,28_ = 0.2, *p* = 0.7; interaction: *F*_1,28_ = 0.2, *p* = 0.7; *frequency*: effect of sex: *F*_1,28_ = 0, *p* = 0.9; effect of ECS: *F*_1,28_ = 2, *p* = 0.2; interaction: *F*_1,28_ = 0.3, *p* = 0.6). As in the test of novel object recognition, ECS‐treated mice moved greater distances across the sample and test phases of the novel place recognition task (Figure 3o; effect of testing phase: *F*_1,29_ = 2.7, *p* = 0.1; effect of ECS: *F*_1,30_ = 4.6, *p* = 0.04; interaction: *F*_1,29_ = 0.1, *p* = 0.8).

Finally, in a test of associative memory, mice were subjected to a test of object‐context memory, where they must distinguish a familiar object in a familiar context, but in a novel configuration (i.e., because the object has never been encountered in that context before). Here, sham‐treated, but not ECS‐treated, mice were able to recognize the novel object‐context configuration (Figure 3p–t). When assessing exploration duration, only sham‐treated mice explored the novel configuration more than the familiar one (effect of object: *F*_1,30_ = 1, *p* = 0.3, effect of ECS: *F*_1,30_ = 0.3, *p* = 0.6, object × ECS interaction: *F*_1,30_ = 7, *p* = 0.01; sham familiar vs. novel: *p* = 0.02, ECS familiar vs. novel: *p* = 0.4). Likewise, analyses of exploration frequency revealed that sham‐treated mice selectively recognized the novel object‐context configuration (effect of object: *F*_1,30_ = 1, *p* = 0.3, effect of ECS: *F*_1,30_ = 1.2, *p* = 0.3, object × ECS interaction: *F*_1,30_ = 9, *p* = 0.005; sham novel vs. familiar: *p* = 0.01, ECS novel vs. familiar: *p* = 0.3). Analyses of discrimination indices also revealed selective object‐context memory in the sham‐treated mice (*duration*: effect of sex: *F*_1,28_ = 0.3, *p* = 0.6; effect of ECS: *F*_1,28_ = 6, *p* = 0.02; interaction: *F*_1,28_ = 1.3, *p* = 0.3; *frequency*: effect of sex: *F*_1,28_ = 0, *p* = 0.8; effect of ECS: *F*_1,28_ = 10, *p* = 0.004; interaction: *F*_1,28_ = 0, *p* = 0.9). In this task, there were no differences in total locomotion between sham and ECS mice (Figure 3u; effect of testing phase: *F*_2,27_ = 1.4, *p* = 0.3; effect of ECS: *F*_1,28_ = 0, *p* = 1; interaction: *F*_2,27_ = 1.5, *p* = 0.2).

## 4 Discussion

At least some of the negative effects of ECT on memory and cognition are likely to depend on changes in hippocampal physiology and plasticity. Here we therefore examined how ECS, in mice, impacts relevant properties of hippocampal neurons and circuits. We found that ECS impaired synaptic activation of DG neurons ex vivo and blunted experience‐dependent activation of hippocampal neurons in vivo. Behaviorally, ECS‐treated mice failed to learn object‐context associations, consistent with hippocampal dysfunction (Mumby et al. 2002; Wilson, Langston, et al. 2013).

The DG has been implicated in learning since at least as far back as the first report of long‐term potentiation in the mammalian brain (Bliss and Lomo 1973). Since then, evidence has accumulated which links entorhinal‐DG/CA3 synaptic plasticity, sparse coding, and excitation‐inhibition balance to hippocampal memory processes (Moser et al. 1998; Reagh et al. 2018; Madar et al. 2019; McHugh et al. 2022). Here, we did not observe any changes in intrinsic membrane properties that could influence the excitability of DG neurons, and therefore their recruitment during learning (Mocle et al. 2024). Instead, ECS caused synaptic and circuit‐level changes that reduced perforant path‐DG coupling: using physiologically‐relevant trains of stimuli to examine how DG neurons respond to brief episodes of afferent activation, we found that excitatory cortical input synapses were weaker and less able to induce spiking in postsynaptic DG neurons.

Notably, reduced synaptic activation was only apparent under GABA_A_ blockade. With inhibition intact, synaptic responses were similar between sham and ECS mice, and both groups displayed similar (low) rates of spiking. It is well established that there is exceptionally strong GABAergic suppression of DG activity (Coulter and Carlson 2007; Marín‐Burgin et al. 2012). Therefore, a stronger (direct or indirect) recruitment of GABAergic interneurons in sham mice may have offset their stronger excitatory synaptic inputs, resulting in comparable amounts of synaptic activation in sham and ECS mice when inhibition was intact.

An outstanding question is why EPSP magnitude did not increase in ECS mice when GABA_A_ receptors were blocked. One possibility is that there are additional sources of inhibition that are recruited following ECS. For example, immature granule neurons appear to directly inhibit mature granule neurons via Group 2 metabotropic glutamate receptors (mGluR2) (Luna et al. 2019). Since adult neurogenesis is dramatically increased by ECS (Zhang et al. 2026), there may be a large pool of immature neurons that are recruited to inhibit the mature granule neuron population. Consistent with this, broad optogenetic activation of immature neurons leads to greater mGluR2‐dependent inhibition of mature granule neurons in mice that were treated with ECS (Santiago et al. 2026).

Our findings that ECS reduced excitatory synaptic activation contrasts with previous reports that ECS increases EPSP‐spike coupling in the DG (Imoto et al. 2017) and increases synapse strength, in an LTP‐like fashion (Stewart et al. 1994; Stewart and Reid 2000; Chen et al. 2018). Discrepancies could reflect species (mouse vs. rat) or preparation differences (in vivo vs. ex vivo). Alternatively, they could reflect differences in the ECS‐testing interval. Where previous studies have found synaptic potentiation‐like effects 1 day following chronic ECS, here we found synaptic depression‐like effects several days after chronic ECS (on average 3 days but up to 7 days post‐ECS). Such dynamic physiological changes following ECS may relate to the pattern of behavioral changes in patients, where many of the adverse cognitive side‐effects of ECT resolve in the days and weeks after treatment (Semkovska and McLoughlin 2010; Landry et al. 2020). In any case, the reduced spiking and weaker synaptic transmission is consistent with our in vivo Fos data, as well as a similar report by others (Santiago et al. 2026), that ECS reduces activity in the DG as measured by Fos expression. While sparse codes are thought to be fundamental to DG functions in pattern separation and reducing memory interference (GoodSmith et al. 2017; Cholvin and Bartos 2026), our data suggest that excessive hypoactivity may be detrimental to associative recognition memory processes.

ECT impairs anterograde learning and memory, as measured by recognition memory tests, where patients study a list of items (e.g., words, images, spatial patterns) and are tested on their ability to recognize novel versus familiar items at various times after training (Squire and Miller 1974; Steif et al. 1986; Sackeim et al. 2000; Falconer et al. 2010; Fernie et al. 2014). Generally, recognition memory is most impaired at early timepoints after ECT (minutes to days) and at longer training–testing intervals (hours to days) (Squire and Miller 1974; Steif et al. 1986). However, some forms of recognition memory, such as spatial recognition, are impaired for months after treatment before recovering (Falconer et al. 2010; Fernie et al. 2014). However, due to the limited number of studies and challenges associated with studying memory in patient populations, it remains unclear exactly which types of memory are impaired and which brain regions are involved.

In rodents, recognition memory tests have been adapted to test many aspects of episodic‐like memory, including memory for objects, locations, temporal order, and what‐when‐where‐like associations between some or all of these aspects of sensory experience (Dere et al. 2007). The hippocampus (and/or afferent entorhinal cortex) is often required for successful performance especially at longer delays (Clark et al. 2000), for associative forms of recognition memory that may be more dependent on hippocampal recollection (Li and Chao 2008; Sauvage et al. 2008; Langston and Wood 2010; Wilson, Langston, et al. 2013; Wilson, Watanabe, et al. 2013; Chao et al. 2015), or when training and test phase stimuli have a high degree of feature similarity (Kirwan et al. 2012; Bekinschtein et al. 2013; Kesner et al. 2015; McHugh et al. 2022). In particular, the DG is important for recognition memory tasks that have a higher degree of discriminative difficulty and are likely to depend on its pattern separation functions (Bakker et al. 2008; Bekinschtein et al. 2013, 2014; Kesner et al. 2015). Our behavioral findings therefore suggest that hippocampal‐dependent associative recognition memory may be particularly vulnerable to the anterograde amnestic effects of ECS/ECT since mice were able to remember objects and locations but were unable to associate specific objects with specific contexts.

One challenge associated with studying effects on cognition is that, as a treatment for mood and motor disorders, ECT/ECS may also induce confounding changes in emotion or other non‐mnemonic aspects of performance. Indeed, prior work in rodents has found that ECS variably increases activity and/or reduces anxiety (Gersner et al. 2010; Zhang, Ren, et al. 2021; Lebeau et al. 2022; Santiago et al. 2026). Here we observed no ECS‐induced changes in locomotion or center‐time exploration in the open field habituation session prior to recognition memory testing, suggesting normal levels of activity and anxiety. This lack of effect may be due, in part, to the fact that prior studies have typically employed mouse models of depression (ECS restores activity/anxiety to baseline), whereas our mice were naïve. However, in the object and place recognition tasks we indeed found that ECS mice displayed higher levels of locomotion and in the novel object testing phase ECS mice also explored the objects to a greater extent. Relative exploration of the novel versus familiar stimuli was not different, indicating intact memory. However, we cannot fully rule out the possibility that a potential memory deficit was obscured by greater levels of exploration (i.e., learning) in ECS mice. Equivalent levels of locomotion in the object‐context task, however, suggests that ECS causes a more pure deficit in associative recognition memory.

Although we included both male and female mice, we did not detect sex differences in ECS effects on DG synaptic transmission, experience‐induced Fos expression, or memory. This is compatible with the emerging ECS/ECT literature, in which sex effects appear to be outcome‐ and parameter‐dependent rather than universal. In rodents, there are sex differences in ECS’ antidepressant‐like behavioral efficacy, age sensitivity, and long‐term cognitive outcomes, yet some DG plasticity measures, including neurogenic plasticity and BDNF expression, change similarly in males and females (García‐Cabrerizo et al. 2020; Ledesma‐Corvi and García‐Fuster 2023a, 2023b; Zhang et al. 2026). This is in contrast to repeated transcranial magnetic stimulation (TMS), which induces DG mossy fiber plasticity selectively in male mice (Zhang et al. 2023). This difference between the two stimulation modalities may reflect the strength of the stimulus, where ECS is suprathreshold for eliciting plastic changes in both sexes. In humans, large ECT cohorts generally report similar remission rates in women and men (Blanken et al. 2023), although sex may influence seizure threshold and cognitive side‐effect measures, including retrograde amnesia as measured by autobiographical memory tests (Coffey et al. 1995; Sackeim et al. 2007). Together, these findings suggest that sex may be an important factor to consider when assessing behavioral outcomes, but they also indicate that not all hippocampal endpoints differ by sex.

While this study did not probe causation, our behavioral, electrophysiological, and Fos expression data provide evidence that ECS impairs DG circuitry, which could contribute to the anterograde amnesia in associative memory paradigms. Future experiments that prevent ECS/ECT‐induced changes in specific brain regions, and further probe the nature of the memory deficits, are needed to identify exactly where and how ECS/ECT alters memory.

## Funding

This work was supported by the Canadian Institutes of Health Research; Fondation Brain Canada; Michael Smith Health Research BC; Vancouver Coastal Health Research Institute; Weston Brain Institute; Seedlings Foundation.

## Supporting information


**Data S1:** hipo70142‐sup‐0001‐DataS1.xlsx.

## Data Availability

The data that supports the findings of this study are available in the Supporting Information of this article (“hipo70142‐sup‐0001‐DataS1.xlsx”).
